# Managing a Rivaroxaban Bleed: Understanding the Difficulties in Acute Reversal of the New Oral Anticoagulants through a Case Report

**DOI:** 10.1155/2014/548272

**Published:** 2014-11-13

**Authors:** Naveen Nannapaneni, Robby Singh, Paulina Mckay, Marwan Al-Hajeili

**Affiliations:** ^1^Department of Internal Medicine, Detroit Receiving Hospital/University Health Center, Suite 2E, 4201 St. Antoine Street, Detroit, MI 48201, USA; ^2^Pharmacy Department, Detroit Medical Center, Detroit, MI, USA; ^3^Department of Internal Medicine, King Abdulaziz University, Jeddah, Saudi Arabia

## Abstract

With the arrival of a new generation of oral anticoagulants significant burdens associated with warfarin's use on both the patient and the healthcare system have been alleviated. Nevertheless, a shortfall exists in regard to an agent or protocol for reversal of these new anticoagulants in the setting of an acute bleed. Our case of a patient presenting to the hospital with a vaginal bleed while on rivaroxaban highlights the difficulty in management without a clear protocol or agent for reversal of anticoagulation.

## 1. Introduction

For decades the mainstay of outpatient anticoagulation has been warfarin, an inhibitor of hepatic synthesis of vitamin K dependent coagulation factors [1]. Annual prescriptions for warfarin number in the tens of millions as it is used for a multitude of indications including treatment/prevention of venous thromboembolism and prophylaxis in nonvalvular atrial fibrillation and cardiac-valve replacement [1]. It requires close monitoring for its narrow therapeutic window, prolonged onset/offset, and variable response, all of which must be managed in the setting of several medication interactions, making it a problematic drug for both patient and physician [1]. With the arrival of a new generation of oral anticoagulants—direct factor Xa inhibitors rivaroxaban (Xarelto) and dabigatran (Pradaxa) and the direct thrombin inhibitor apixaban (Eliquis)—many of these burdens on both the patient and the healthcare system have been alleviated. These medications do not require close monitoring of anticoagulation parameters; they have fewer drug interactions through cytochrome enzymes, and no dietary restrictions of food, making them more convenient while still maintaining efficacy on par with, or better than, warfarin [2–4]. They have since gained FDA approval for additional indications [5] which will undoubtedly increase the number of users in the years to come. As more physicians begin to adopt these oral anticoagulants into their practice a concurrent increase in medication sequelae is to be expected, the most important of which is acute bleeding. There is no agent for immediate reversal of the new oral anticoagulants in the status quo [6] and the management of such cases can be difficult, as exhibited with our patient.

## 2. Case Presentation

A 39-year-old woman with a history of schizoaffective disorder and bilateral pulmonary emboli being treated with rivaroxaban presented to the Emergency Room (ER) with a two-week history of vaginal bleeding. She endorsed fatigue, dyspnea, and lightheadedness while associating abdominal cramping and low back pain with the bleeding. She reported no prior history of abnormal menstrual bleeding. She stated she was transitioned from warfarin to rivaroxaban approximately six weeks ago, with hospital records indicating a switch to rivaroxaban 20 mg daily was made due to concerns of noncompliance with warfarin monitoring. Physical exam revealed blood pressure 122/70 mmHg, heart rate 70 beats per minute, respiratory rate 18 breaths per minute with an oxygen saturation of 99% on room air, and a temperature of 36°C. Pelvic exam showed fresh blood and clots in the vaginal vault, and after they were cleared, bleeding from a closed external cervical os was identified. Laboratory investigations showed her hemoglobin on presentation was 6.8 g/dL, 4.0 g/dL below routine lab work done one month before, platelet count 266,000/*μ*L, prothrombin time 14.1 seconds, activated partial thromboplastin time 27.2 seconds, and INR 1.3.

In the ER she was started on intravenous fluids and given 1,800 units of intravenous activated prothrombin complex concentrate (PCC) per ER protocol to attempt reversal of anticoagulation. Upon admission hematology was consulted and their recommendations of holding rivaroxaban, transfusing two units of packed red blood cells, and discontinuing the PCC therapy, due to the increased risk of thrombosis, were followed. Through the next day the patient continued to bleed with her hemoglobin dropping from 8.3 g/dL after the initial transfusion to 5.7 g/dL and attempts to stop the bleeding with 2.5 mg intravenous conjugated estrogen recommended by gynecology were unsuccessful. By the third day of admission the patient had received an additional 4 units of packed red blood cells with improvement in the hemoglobin to 8.3 g/dL. Notably, repeat coagulation studies revealed a prothrombin time of 14.6 seconds, activated partial thromboplastin time of 21.5 seconds, and INR 1.3 (Table 1). Gynecology attempted balloon tamponade which was also unsuccessful in inducing termination of bleeding. After discussing the implication to her fertility, the patient was agreeable to a dilation and curettage with endometrial ablation the next day which resulted in cessation of bleeding and stabilization of hemoglobin to 9.5 g/dL after one additional unit of packed red blood cells was transfused. She was discharged on fondaparinux with hematology follow-up.

## 3. Discussion

In summary, our patient with a history of unprovoked pulmonary emboli being treated with rivaroxaban presented with endometrial bleeding necessitating attempts at bleeding termination including unsuccessful mechanical compression and PCC, ultimately requiring endometrial ablation. The inciting cause for her endometrial bleed was likely an underlying anatomical abnormality that was provoked by the use of rivaroxaban as there are no reports of endometrial bleeding being a specific side effect of the medication. Further complicating her case was the concern of appropriate medication dosing as the patient provided varying reports to physicians as to the frequency she was taking the pills, informing some that she was taking it once daily and reporting to others that she was taking it twice a day, which could have led to supratherapeutic levels of anticoagulation.

The acute management of a bleed while on rivaroxaban is a challenge for any physician. In our case, the patient was given PCC based on the hospital's ER protocol. It was designed based on a study which reported efficacy of PCC to reverse the anticoagulant effect of rivaroxaban [7]. Since publication, this study has been met with some skepticism in regard to PCC being an antidote for reversal. First, it was conducted in nonbleeding healthy subjects and used endogenous thrombin potential and prothrombin times to gauge reversal, both of which are suboptimal methods of monitoring rivaroxaban [8]. The sensitivity of prothrombin time varies based on the reagent used and there is no standardized conversion to use across laboratories and institutions [8]. Moreover, it has been shown in animal models that an improvement in these coagulation studies did not always correlate with a reduction in clinical bleeding [9] and that the improvement observed in lab parameters was not always sustained [10]. Its availability and cost are additional prohibitive concerns limiting its widespread use [11].

Further relevant to our case, the use of PCC is also associated with an increased risk of thrombotic complications such as venous thromboembolism, disseminated intravascular coagulation, microvascular thrombosis, and myocardial infarction [12–14] which was a significant concern given our patient's history of underlying unprovoked pulmonary emboli. Without a workup for a potential underlying thrombophilia the use of PCC may have increased her risk for any of these ramifications. This concern was shared by the hematology consultants who felt the risk of additional clot burden for the patient did not justify continuing the PCC started in the ER. This risk of thrombosis needs to be addressed on a case-by-case basis depending on the indication for anticoagulation. For example, a different threshold for the clinician's concern may exist for a patient on anticoagulation for nonvalvular atrial fibrillation versus a patient with an underlying hypercoagulable state being treated for a venous thromboembolism. The use of PCC for a major bleed remains a topic for debate and at present it has not gained FDA approval as an agent for acute reversal.

In contrast, bleeding with warfarin is managed with a clear protocol for emergent reversal based on the INR as a result of extensive research into this complication [15]. Therapies ranging from holding doses of warfarin, vitamin K replacement, fresh frozen plasma (FFP), or PCC are used depending on the INR and clinical setting. Recent trials using current reversal modalities for rivaroxaban have not yielded promising results. Interventions such as FFP and recombinant activated factor VII have been studied and shown to be ineffective [16]. The quantity of clotting factors in FFP is relatively miniscule–one dose of PCC has the amount of clotting factors found in 8–16 units of FFP—and thawing and transfusing such large volumes would be impractical in an acute setting. As a result, current therapies entail supportive management including red blood cell transfusions and local mechanical compression for minor bleeds. Due to its shorter half life, 9–13 hours for rivaroxaban versus 20–60 hours for warfarin, holding the medication will likely suffice for such cases. Surgical intervention based on cause and severity should also be considered, especially in major bleeds.

Attempts at developing a recombinant factor Xa, which would allow for immediate reversal by providing a competitive binding site for the Xa inhibitor and permit normal progression through the coagulation cascade, are in process and would likely provide an idyllic solution [17]. Until that time, additional investigation into the utility of PCC in actively bleeding patients on rivaroxaban is warranted. Its use must be thoroughly considered on a case-by-case basis to weigh the known risks and the potential benefits. With the ever increasing number of patients using these new oral anticoagulants the frequency of acute bleeds similar to our case encountered by physicians will continue to rise and developing a medication and protocol for immediate reversal is of paramount importance to prevent morbidity and mortality associated with new oral anticoagulant-induced bleeds.

## Figures and Tables

**Table 1:** Timeline of hospitalization.

Hospital day no.	1	2	3	4	5

Hgb (g/dL)	6.8, 8.3, 7.2, 5.7	5.1, 9, 8.3	9.3, 9.7, 9.2	8.2, 7.8, 9.3	9.5

PT (seconds)	14.1	14.6	12.6		

aPTT (seconds)	27.2	21.5	21.9		

INR	1.3	1.3	1.1		

Intervention	1,800 units of prothrombin complex concentrate, intravenous fluids, and 2 units of packed red blood cells	2.5 mg conjugated estrogen and 2 units of packed red blood cells	Balloon tamponade and 2 units of packed red blood cells	Endometrial ablation and 1 unit of packed red blood cells	Discharge

Hgb: hemoglobin, PT: prothrombin time, aPTT: activated partial thromboplastin time, INR: international normalized ratio.
